# 2,4-Dichloro­phenyl benzoate

**DOI:** 10.1107/S1600536809011763

**Published:** 2009-04-02

**Authors:** B. Thimme Gowda, Miroslav Tokarčík, Jozef Kožíšek, P. A. Suchetan, Hartmut Fuess

**Affiliations:** a Department of Chemistry, Mangalore University, Mangalagangotri 574 199, Mangalore, India; b Faculty of Chemical and Food Technology, Slovak Technical University, Radlinského 9, SK-812 37 Bratislava, Slovak Republic; c Institute of Materials Science, Darmstadt University of Technology, Petersenstrasse 23, D-64287 Darmstadt, Germany

## Abstract

The crystal structure of the title compound, C_13_H_8_Cl_2_O_2_, resembles those of 2,3-dichloro­phenyl benzoate, 2,4-dimethyl­phenyl benzoate and other aryl benzoates, with similar bond parameters. The plane of central –C(=O)—O– group is inclined at the angle of 9.1 (2)° with respect to the benzoate ring. The two aromatic rings make a dihedral angle of 47.8 (1)°. In the crystal structure there are no classical hydrogen bonds. The mol­ecules in the structure are packed into chains diagonally in the *bc* plane.

## Related literature

For the preparation of the compound, see: Nayak & Gowda (2009 ▶); For related structures, see: Gowda *et al.* (2007 ▶, 2008 ▶, 2009 ▶).
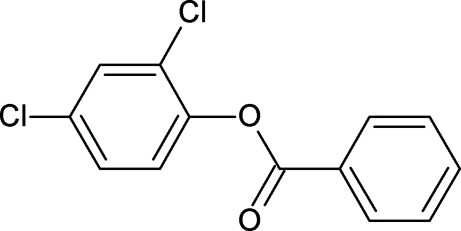


## Experimental

### 

#### Crystal data

C_13_H_8_Cl_2_O_2_
*M* *_r_* = 267.09
Orthorhombic, 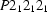

*a* = 3.9722 (1) Å
*b* = 11.9458 (3) Å
*c* = 24.9407 (5) Å
*V* = 1183.46 (5) Å^3^
*Z* = 4
Mo *K*α radiation
μ = 0.53 mm^−1^
*T* = 295 K
0.47 × 0.11 × 0.10 mm

#### Data collection

Oxford Diffraction Xcalibur diffractomenter with a Ruby (Gemini Mo) detector
Absorption correction: multi-scan (*CrysAlis RED*; Oxford Diffraction, 2009 ▶) *T*_min_ = 0.751, *T*_max_ = 0.943
20045 measured reflections
2202 independent reflections
1961 reflections with *I* > 2σ(*I*)
*R*_int_ = 0.027

#### Refinement

*R*[*F* ^2^ > 2σ(*F* ^2^)] = 0.023
*wR*(*F* ^2^) = 0.059
*S* = 1.03
2202 reflections
160 parameters
2 restraints
H atoms treated by a mixture of independent and constrained refinement
Δρ_max_ = 0.13 e Å^−3^
Δρ_min_ = −0.15 e Å^−3^
Absolute structure: Flack (1983 ▶), 861 Friedel pairs
Flack parameter: 0.02 (5)

### 

Data collection: *CrysAlis CCD* (Oxford Diffraction, 2009 ▶); cell refinement: *CrysAlis RED* (Oxford Diffraction, 2009 ▶); data reduction: *CrysAlis RED*; program(s) used to solve structure: *SHELXS97* (Sheldrick, 2008 ▶); program(s) used to refine structure: *SHELXL97* (Sheldrick, 2008 ▶); molecular graphics: *ORTEP-3* (Farrugia, 1997 ▶) and *DIAMOND* (Brandenburg, 2002 ▶); software used to prepare material for publication: *SHELXL97*, *PLATON* (Spek, 2009 ▶) and *WinGX* (Farrugia, 1999 ▶).

## Supplementary Material

Crystal structure: contains datablocks I, global. DOI: 10.1107/S1600536809011763/bt2919sup1.cif

Structure factors: contains datablocks I. DOI: 10.1107/S1600536809011763/bt2919Isup2.hkl

Additional supplementary materials:  crystallographic information; 3D view; checkCIF report

## Figures and Tables

**Fig. 1.**
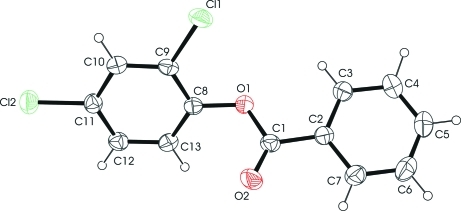
Molecular structure of the title compound showing the atom labelling scheme. Displacement ellipsoids are drawn at the 30% probability level. H atoms are represented as small spheres of arbitrary radii.

**Fig. 2.**
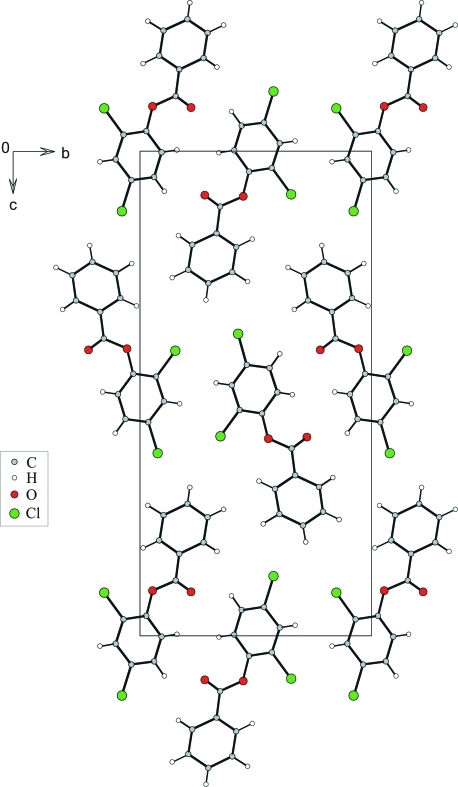
The packing of the title compound viewed down the a axis.

## supplementary crystallographic information

### Comment

In the present work, as a part of studying the substituent effects on the crystal structures of aryl benzoates (Gowda *et al.*, 2007, 2008, 2009), the structure of 2,4-dichlorophenyl benzoate   has been determined. The structure   (Fig. 1) is similar to those of 2,3-dichlorophenylbenzoate (Gowda *et al.*, 2007), 2,4-dimethylphenyl benzoate (Gowda *et al.*, 2008) and other aryl benzoates (Gowda *et al.*, 2009). The two aromatic rings  make a dihedral angle of 47.8 (1)°. The plane of the –C(=O)–O– group is inclined at an angle of 9.1 (2)° to the benzoate ring. In the crystal structure, there are no classical hydrogen bonds. The molecules in the structure are packed into chains as viewed down the *bc* plane (Fig. 2).

### Experimental

The title compound was prepared according to a literature method (Nayak & Gowda, 2009). The purity of the compound was checked by determining its melting point. It was characterized by recording its infrared and NMR spectra (Nayak & Gowda, 2009). Single crystals of the title compound were obtained by slow evaporation of its ethanol solution. The X-ray diffraction studies were made at room temperature.

### Refinement

H atoms were positioned geometrically and refined using a riding model with C—H distances of 0.93 Å, except for H atoms bound to C4 and C5, which were subject to the restraint on the C—H distance (set to 0.95 (4) Å). This measure improved the anisotropic displacement parameters of the atoms C4, C5 and enabled to remove the alert_C regarding the Hirshfeld-test. All H atoms were refined with *U*_iso_(H) = 1.2 times *U*_eq_(C).

### Crystal data

**Table:**

C_13_H_8_Cl_2_O_2_	*F*(000) = 544
*M**_r_* = 267.09	*D*_x_ = 1.499 Mg m^−^^3^
Orthorhombic, *P*2_1_2_1_2_1_	Mo *K*α radiation, λ = 0.71073 Å
Hall symbol: P 2ac 2ab	Cell parameters from 10923 reflections
*a* = 3.9722 (1) Å	θ = 3.3–29.5°
*b* = 11.9458 (3) Å	µ = 0.53 mm^−^^1^
*c* = 24.9407 (5) Å	*T* = 295 K
*V* = 1183.46 (5) Å^3^	Needle, colourless
*Z* = 4	0.47 × 0.11 × 0.10 mm

### Data collection

**Table:**

Oxford Diffraction Xcalibur diffractomenter with a Ruby (Gemini Mo) detector	2202 independent reflections
graphite	1961 reflections with *I* > 2σ(*I*)
Detector resolution: 10.434 pixels mm^-1^	*R*_int_ = 0.027
ω scans	θ_max_ = 25.5°, θ_min_ = 3.3°
Absorption correction: multi-scan (*CrysAlis RED*; Oxford Diffraction, 2009)	*h* = −4→4
*T*_min_ = 0.751, *T*_max_ = 0.943	*k* = −14→14
20045 measured reflections	*l* = −30→30

### Refinement

**Table:**

Refinement on *F*^2^	Secondary atom site location: difference Fourier map
Least-squares matrix: full	Hydrogen site location: inferred from neighbouring sites
*R*[*F*^2^ > 2σ(*F*^2^)] = 0.023	H atoms treated by a mixture of independent and constrained refinement
*wR*(*F*^2^) = 0.059	*w* = 1/[σ^2^(*F*_o_^2^) + (0.0343*P*)^2^ + 0.0694*P*] where *P* = (*F*_o_^2^ + 2*F*_c_^2^)/3
*S* = 1.03	(Δ/σ)_max_ < 0.001
2202 reflections	Δρ_max_ = 0.13 e Å^−^^3^
160 parameters	Δρ_min_ = −0.15 e Å^−^^3^
2 restraints	Absolute structure: Flack (1983), 861 Friedel pairs
Primary atom site location: structure-invariant direct methods	Flack parameter: 0.02 (5)

### Special details

**Table:**

Geometry. All e.s.d.'s (except the e.s.d. in the dihedral angle between two l.s. planes) are estimated using the full covariance matrix. The cell e.s.d.'s are taken into account individually in the estimation of e.s.d.'s in distances, angles and torsion angles; correlations between e.s.d.'s in cell parameters are only used when they are defined by crystal symmetry. An approximate (isotropic) treatment of cell e.s.d.'s is used for estimating e.s.d.'s involving l.s. planes.
Refinement. Refinement of *F*^2^ against ALL reflections. The weighted *R*-factor *wR* and goodness of fit *S* are based on *F*^2^, conventional *R*-factors *R* are based on *F*, with *F* set to zero for negative *F*^2^. The threshold expression of *F*^2^ > σ(*F*^2^) is used only for calculating *R*-factors(gt) *etc*. and is not relevant to the choice of reflections for refinement. *R*-factors based on *F*^2^ are statistically about twice as large as those based on *F*, and *R*- factors based on ALL data will be even larger.

### Fractional atomic coordinates and isotropic or equivalent isotropic displacement parameters (Å^2^)

**Table:**

	*x*	*y*	*z*	*U*_iso_*/*U*_eq_	
C1	0.4499 (4)	0.34482 (14)	0.11405 (6)	0.0475 (4)	
C2	0.5855 (4)	0.32994 (13)	0.16904 (6)	0.0454 (4)	
C3	0.7373 (5)	0.41579 (15)	0.19708 (7)	0.0532 (4)	
H3	0.7648	0.4856	0.1811	0.064*	
C4	0.8484 (6)	0.39790 (19)	0.24887 (7)	0.0649 (5)	
H4	0.947 (5)	0.4659 (16)	0.2689 (8)	0.078*	
C5	0.8127 (6)	0.29517 (19)	0.27219 (8)	0.0671 (5)	
H5	0.884 (6)	0.2847 (17)	0.3063 (8)	0.081*	
C6	0.6657 (6)	0.20918 (18)	0.24438 (8)	0.0709 (6)	
H6	0.6437	0.1393	0.2604	0.085*	
C7	0.5496 (5)	0.22556 (15)	0.19272 (7)	0.0603 (5)	
H7	0.4485	0.1672	0.174	0.072*	
C8	0.4313 (4)	0.47198 (13)	0.04142 (6)	0.0439 (4)	
C9	0.2560 (4)	0.56988 (13)	0.03415 (6)	0.0436 (4)	
C10	0.1619 (4)	0.60433 (13)	−0.01650 (6)	0.0466 (4)	
H10	0.0463	0.6712	−0.0215	0.056*	
C11	0.2437 (4)	0.53692 (13)	−0.05937 (6)	0.0457 (4)	
C12	0.4179 (4)	0.43887 (13)	−0.05300 (6)	0.0489 (4)	
H12	0.4704	0.3947	−0.0825	0.059*	
C13	0.5148 (4)	0.40651 (14)	−0.00208 (7)	0.0489 (4)	
H13	0.636	0.3407	0.0028	0.059*	
O1	0.5392 (3)	0.44515 (9)	0.09308 (4)	0.0522 (3)	
O2	0.2813 (4)	0.27886 (11)	0.09054 (5)	0.0749 (4)	
Cl1	0.14820 (14)	0.65271 (4)	0.088572 (17)	0.06409 (15)	
Cl2	0.11922 (13)	0.57737 (4)	−0.123305 (17)	0.06442 (15)	

### Atomic displacement parameters (Å^2^)

**Table:**

	*U*^11^	*U*^22^	*U*^33^	*U*^12^	*U*^13^	*U*^23^
C1	0.0547 (10)	0.0419 (8)	0.0460 (8)	−0.0075 (9)	0.0057 (8)	−0.0025 (7)
C2	0.0474 (9)	0.0440 (9)	0.0450 (8)	−0.0005 (8)	0.0081 (7)	−0.0007 (7)
C3	0.0588 (10)	0.0515 (10)	0.0493 (9)	−0.0038 (9)	0.0020 (8)	0.0003 (8)
C4	0.0657 (12)	0.0814 (14)	0.0477 (10)	−0.0034 (12)	−0.0026 (9)	−0.0054 (9)
C5	0.0699 (13)	0.0840 (14)	0.0474 (10)	0.0095 (12)	0.0021 (10)	0.0095 (11)
C6	0.0816 (14)	0.0657 (12)	0.0654 (12)	0.0070 (13)	0.0145 (11)	0.0207 (10)
C7	0.0742 (13)	0.0497 (10)	0.0571 (11)	−0.0025 (10)	0.0093 (10)	0.0030 (8)
C8	0.0467 (9)	0.0394 (8)	0.0455 (8)	−0.0080 (8)	−0.0019 (7)	0.0002 (6)
C9	0.0466 (9)	0.0371 (8)	0.0470 (9)	−0.0076 (7)	0.0047 (7)	−0.0072 (7)
C10	0.0479 (9)	0.0401 (8)	0.0517 (9)	0.0020 (8)	0.0019 (8)	−0.0005 (7)
C11	0.0460 (9)	0.0485 (9)	0.0425 (8)	−0.0045 (8)	0.0005 (7)	0.0036 (7)
C12	0.0518 (10)	0.0482 (9)	0.0468 (9)	0.0031 (9)	0.0063 (8)	−0.0060 (7)
C13	0.0516 (9)	0.0411 (9)	0.0541 (9)	0.0043 (8)	0.0012 (8)	0.0009 (7)
O1	0.0667 (8)	0.0424 (6)	0.0475 (6)	−0.0113 (6)	−0.0117 (6)	0.0031 (5)
O2	0.1069 (11)	0.0634 (8)	0.0546 (7)	−0.0411 (8)	−0.0074 (8)	−0.0019 (6)
Cl1	0.0868 (3)	0.0509 (2)	0.0545 (2)	−0.0017 (2)	0.0102 (2)	−0.0149 (2)
Cl2	0.0754 (3)	0.0715 (3)	0.0464 (2)	0.0074 (3)	−0.0040 (2)	0.0055 (2)

### Geometric parameters (Å, °)

**Table:**

C1—O2	1.189 (2)	C7—H7	0.93
C1—O1	1.355 (2)	C8—C9	1.373 (2)
C1—C2	1.484 (2)	C8—C13	1.378 (2)
C2—C3	1.380 (2)	C8—O1	1.3951 (19)
C2—C7	1.387 (2)	C9—C10	1.380 (2)
C3—C4	1.382 (3)	C9—Cl1	1.7334 (15)
C3—H3	0.93	C10—C11	1.378 (2)
C4—C5	1.365 (3)	C10—H10	0.93
C4—H4	1.031 (19)	C11—C12	1.370 (2)
C5—C6	1.370 (3)	C11—Cl2	1.7380 (16)
C5—H5	0.905 (19)	C12—C13	1.382 (2)
C6—C7	1.382 (3)	C12—H12	0.93
C6—H6	0.93	C13—H13	0.93
			
O2—C1—O1	122.91 (15)	C2—C7—H7	120.3
O2—C1—C2	125.52 (15)	C9—C8—C13	120.10 (14)
O1—C1—C2	111.57 (13)	C9—C8—O1	118.25 (13)
C3—C2—C7	119.82 (16)	C13—C8—O1	121.53 (14)
C3—C2—C1	122.54 (14)	C8—C9—C10	120.81 (14)
C7—C2—C1	117.63 (15)	C8—C9—Cl1	120.54 (12)
C2—C3—C4	119.89 (17)	C10—C9—Cl1	118.65 (13)
C2—C3—H3	120.1	C11—C10—C9	118.18 (15)
C4—C3—H3	120.1	C11—C10—H10	120.9
C5—C4—C3	120.3 (2)	C9—C10—H10	120.9
C5—C4—H4	122.8 (11)	C12—C11—C10	121.94 (15)
C3—C4—H4	116.8 (11)	C12—C11—Cl2	119.22 (13)
C4—C5—C6	120.16 (19)	C10—C11—Cl2	118.85 (12)
C4—C5—H5	119.4 (14)	C11—C12—C13	119.11 (15)
C6—C5—H5	120.4 (14)	C11—C12—H12	120.4
C5—C6—C7	120.52 (18)	C13—C12—H12	120.4
C5—C6—H6	119.7	C8—C13—C12	119.85 (16)
C7—C6—H6	119.7	C8—C13—H13	120.1
C6—C7—C2	119.33 (19)	C12—C13—H13	120.1
C6—C7—H7	120.3	C1—O1—C8	118.64 (12)
			
O2—C1—C2—C3	−170.23 (18)	O1—C8—C9—Cl1	−4.3 (2)
O1—C1—C2—C3	9.2 (2)	C8—C9—C10—C11	1.0 (2)
O2—C1—C2—C7	8.5 (3)	Cl1—C9—C10—C11	−178.90 (13)
O1—C1—C2—C7	−172.04 (15)	C9—C10—C11—C12	−0.9 (2)
C7—C2—C3—C4	−1.0 (3)	C9—C10—C11—Cl2	178.61 (13)
C1—C2—C3—C4	177.77 (17)	C10—C11—C12—C13	0.0 (2)
C2—C3—C4—C5	0.9 (3)	Cl2—C11—C12—C13	−179.58 (13)
C3—C4—C5—C6	−0.2 (3)	C9—C8—C13—C12	−0.8 (3)
C4—C5—C6—C7	−0.5 (3)	O1—C8—C13—C12	−176.67 (15)
C5—C6—C7—C2	0.4 (3)	C11—C12—C13—C8	0.9 (3)
C3—C2—C7—C6	0.3 (3)	O2—C1—O1—C8	−0.6 (3)
C1—C2—C7—C6	−178.49 (18)	C2—C1—O1—C8	179.94 (14)
C13—C8—C9—C10	−0.2 (2)	C9—C8—O1—C1	125.12 (16)
O1—C8—C9—C10	175.80 (14)	C13—C8—O1—C1	−59.0 (2)
C13—C8—C9—Cl1	179.76 (13)		
